# Exploring Injectable Scaffolded Spheroids for Nucleus Pulposus Therapy in Degenerated Intervertebral Discs

**DOI:** 10.1021/acsami.5c24306

**Published:** 2026-02-16

**Authors:** Rathina Vel Balasubramanian, Marcia Muerner, Oliver Kopinski-Grünwald, Sibylle Grad, Julia Fernández-Pérez, Aleksandr Ovsianikov

**Affiliations:** † 3D Printing and Biofabrication Group, Institute of Materials Science and Technology, 30148 Technische Universität Wien, Vienna 1040, Austria; ‡ Austrian Cluster for Tissue Regeneration, Vienna 1200, Austria; § 161930 AO Research Institute Davos, Clavadelerstrasse 8, Davos 7270, Switzerland; ∥ Federal Institute of Technology Zurich, Zurich 8092, Switzerland

**Keywords:** microscaffold, scaffolded spheroids, intervertebral disc degeneration, tissue engineering, high-resolution 3D printing, two-photon polymerization, human bone-marrow derived mesenchymal stem sells

## Abstract

Cell-based therapies for intervertebral disc degeneration (IVDD) treatment face significant challenges, including cell damage from injection-induced shear stress and poor survival in the harsh, nutrient-depleted microenvironment of the intervertebral disc. To overcome these challenges, we developed scaffolded spheroids (S-SPH) by integrating human bone marrow-derived mesenchymal stem cell (hBMSC) spheroids (SPH) into microscaffolds (MS) produced via high-resolution 3D printing, thereby forming injectable tissue-building blocks. We optimized cell seeding density (∼2000 cells/spheroid) and MS fabrication parameters and induced nucleus pulposus (NP)-like differentiation using growth differentiation factor-5 (GDF5) under both normoxic and hypoxic, low-glucose conditions mimicking a healthy *in vivo*-like environment. S-SPH maintained high cell viability and produced abundant extracellular matrix under both culture conditions. They also upregulated key NP markers, including aggrecan (ACAN), keratin-18 (KRT18), and hypoxia-inducible factor-1α (HIF1α), which indicated successful NP-like differentiation. They also exhibited improved compressive properties approaching those of native human IVD and retained structural integrity and cell viability following injection through a 26G needle. When differentiated into an NP-like phenotype, S-SPH fused and retained a high viability upon injection *in vitro*. These results demonstrate that S-SPH provide a promising *in vitro* strategy for NP regeneration, warranting further preclinical evaluation for IVDD therapy.

## Graphical abstract

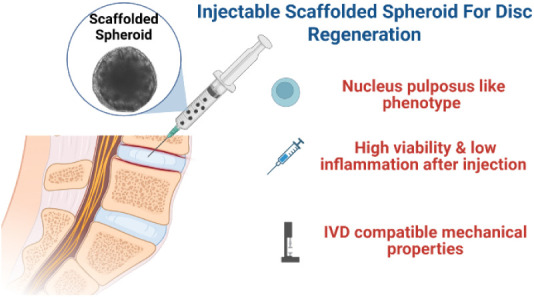

## 1 Introduction

The intervertebral disc (IVD) consists of a central, proteoglycan-rich nucleus pulposus (NP) surrounded by the collagenous annulus fibrosus (AF) and is capped by cartilaginous end plates that anchor the disc to the vertebral bodies.^1^ The hydrated structure of the NP primarily consists of glycosaminoglycans (GAG) and collagen type II, functioning as a shock absorber that disperses hydrostatic pressure during compression.^2^ Intervertebral disc degeneration (IVDD) is the primary cause of low back pain, affecting approximately 12% of the global population. Although IVDD is often age-related, factors such as excessive mechanical loads, injuries, poor nutrient supply, and genetic predispositions can accelerate its onset.^3^ This results in the loss of the proteoglycan-rich matrix, cell senescence, and disruption of the AF, leading to disc height loss, nerve compression, and pain.^3^

The severity of the disease drives the development of future therapeutic approaches for IVDD, although these strategies remain investigational and are not yet in standard clinical use. Current treatments are typically limited to physical therapy, pain medication, and surgery in severe cases. However, research in regenerative medicine proposes a tiered strategy.^4,5^ In the early stages of IVDD, discs retain structural integrity and contain functional cells, which would be utilized by biomolecular strategies such as targeted growth factor delivery or gene therapy to promote tissue repair.^4^ For moderate degeneration, where cell populations diminish and the matrix deteriorates, biomaterial scaffolds offer structural support while activating the remaining cells.^5^ In advanced cases, such as spinal stenosis with disc herniation, cell-based therapies are necessary to repopulate damaged tissue, often used in combination with biomaterials to rebuild functional disc structure.^5^ This tiered strategy aligns treatments with biological changes at each stage, prioritizing cell activation, structural reinforcement, or tissue replacement as required.^4,5^

The primary method for cell-based therapy involves either the injection of cells in saline solution or their combination with soft hydrogels.^4^ However, when cells are injected through a fine-gauge needle or catheter, they face high shear forces that can damage cell membranes and reduce viability.^4^ Consequently, injected cell suspensions often suffer from significant cell loss, undermining therapeutic efficacy.^6^ Upon entering the disc, cells are exposed to low oxygen levels, high osmolarity, and mechanical compression. In the absence of adequate support, they may undergo cell death/apoptosis or differentiate aberrantly.^7^ Pure cell suspensions or soft hydrogels provide little protection from these stresses.^8^ Prior studies indicate that encapsulating cells in stiff or viscoelastic carriers can enhance cell survival by mitigating shear stress. For instance, shear-thinning hydrogels have been shown to protect cells during injection.^9^ However, many carriers do not provide the precise NP-like microenvironments needed for functional regeneration. Therefore, it is important to develop injectable carriers that preserve cell viability during injection and support NP-like differentiation postdelivery. Ideally, such a construct would provide mechanical protection to the cells while allowing the diffusion of nutrients and signaling molecules, thereby promoting the production of the GAG-rich matrix characteristic of healthy NP.

Many researchers are exploring composite microtissues or encapsulation carriers to improve cell-based therapies for IVDD.^10^ For example, spheroids (SPH), three-dimensional aggregates of cells, have been shown to enhance cell–cell interactions and matrix production, fostering a more NP-like extracellular environment.^11^ SPH embody key aspects of native tissue architecture and have emerged as a promising building block for IVD regeneration.^12^ SPH-based approaches enhance cell–cell and cell–matrix interactions, promoting improved extracellular matrix (ECM) production and phenotypic stability compared to monolayer cultures.^10–12^ This strategy has also been investigated for recovering disc height and restoring the ECM by leveraging the inherent properties of SPH, such as their anti-inflammatory effects and ability to mimic tissue-level architecture.^13^ However, scaffold-free SPH face challenges related to mechanical integration and nutrient diffusion, especially when delivered *in vivo*.^14^

Two-photon polymerization (2PP) is a high-resolution additive manufacturing technique that enables the fabrication of complex three-dimensional microstructures with submicron spatial resolution.^15^ The implementation of resonant scanner technology into the 2PP system has recently increased fabrication speed by at least 10-fold,^16^ enabling the large-scale production of polycaprolactone (PCL)-based microscaffolds (MS) with thin struts (approximately 35 μm).^17^ These MS provide tailored mechanical properties and can be surface-modified to promote cell attachment and ECM retention.^15,18^ Building on our group’s recent work, we have enhanced the SPH-based strategy by integrating microscaffolds to create a more robust, mechanically supported microtissue unit known as scaffolded spheroids (S-SPH).^15,18^ We address the compaction property of multiple SPH by producing a more stable structure. The modular tissue demonstrated high viability and differentiation potential using human adipose-derived stem cells (hASC) while maintaining the ability to self-assemble into large tissue structures without external manipulation.^18^

Using human bone marrow-derived stromal cells (hBMSC) for IVDD, particularly for NP regeneration, is challenging due to the harsh microenvironment of the NP, characterized by inflammation, low nutrient levels, and acidity.^19^ A recent report from Mesoblast’s phase II/III clinical trial indicated that their hBMSC-based product helped reduce some pain-related symptoms of IVDD.^20^ However, MRI scans showed no evidence of structural tissue regeneration.^20^ As a result, numerous studies have focused on promoting the differentiation of hBMSC into NP-like cells.^21^ Environmental conditions, such as the oxygen concentration, can significantly influence this differentiation process. Furthermore, hBMSC have been primed with differentiation factors, regulatory molecules, and transfection agents to better adapt to the IVD environment and assist in the regeneration process.^22,23^ Growth differentiation factor 5 (GDF5) has been frequently used to differentiate hBMSC into NP-like phenotypes. It replaced the previous gold standard, transforming growth factor β1 (TGFβ1).^24^ Several studies have reported that GDF5 promotes the formation of NP-like ECM and upregulates NP-specific genes such as FOXF1, KRT19, and PAX1.^25,26^ Additionally, GDF5 and TGFβ1 have successfully produced fully chondrogenic pellets and may serve as an effective combination for differentiating BMSC into NP-like cells.^27^ However, to date, no study has utilized GDF5 to induce the differentiation of S-SPH for the fabrication of NP-like microtissue units.

Building on these advancements, the present study integrates hBMSC-derived spheroids with 2PP-fabricated MS to create optimized S-SPH for NP-like differentiation. We systematically evaluate parameters including seeding density, mechanical properties, the effects of growth factors (GDF5 *vs* TGFβ1), injectability profile, and healthy *in vivo*-like conditions, with the aim of advancing the development of injectable, mechanically robust microtissue units for effective IVD regeneration (Figure 1).

**Figure 1:**
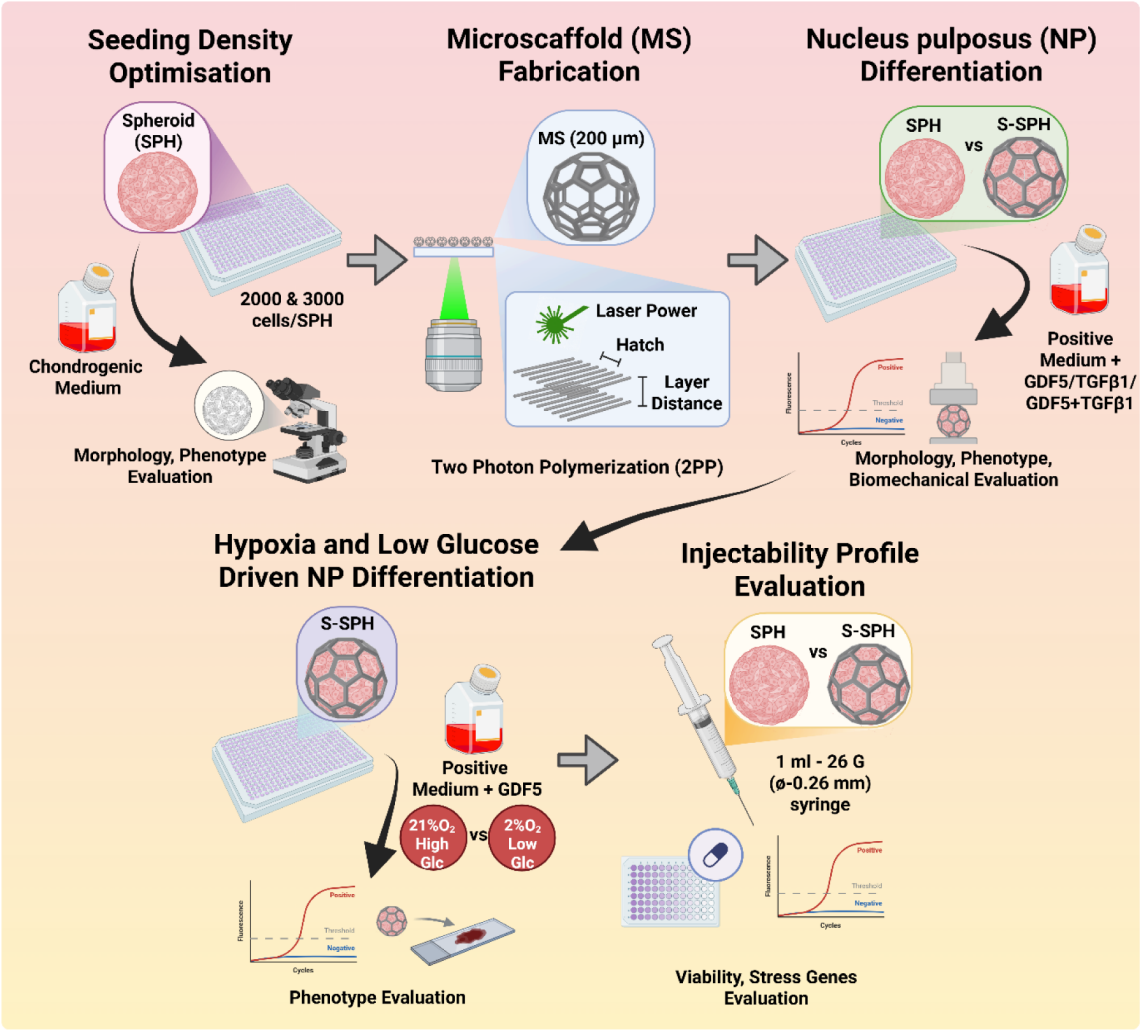
Schematic representation of the workflow of the study. Created in BioRender. Balasubramanian (2026) https://BioRender.com/wkdqh0d.

## 2 Materials and Methods

### 2.1 Optimization of Cell Seeding Density of hBMSC SPH for MS

Human bone marrow was obtained from one donor (male, 24 years old) with ethical approval from the local authorities (Ethik-Kommission der Albert-Ludwigs-Universität Freiburg, EK-326/08) and written consent from the patient undergoing total hip replacement. hBMSC were isolated via density centrifugation separation and plastic adhesion and expanded in Minimum Essential Medium alpha (αMEM) supplemented with 10% Fetal Bovine Serum (FBS), 1% Penicillin–Streptomycin, and 5 ng/mL recombinant human basic fibroblast growth factor (bFGF) at 37 °C, 5% CO_2_. Further, hBMSC (P3–P5, 1 donor, 24Y male) were expanded using αMEM (Gibco: 22571-020) supplemented with 10% FBS (Gibco: 10500-064), 1% Penicillin–Streptomycin 100× (Sigma: P433), and 5 ng/mL of bFGF (Peprotech:100-18B, 10 μg). Once the cells reached 70–80% confluency, they were seeded in low-adhesion 384-well plates at two different cell densities: 2000 and 3000 cells/spheroid. The expansion medium was used for 2 days to culture the spheroids. After 2 days (Day 0), the SPH were divided into two groups based on the medium composition: Expansion Medium (EM) and Positive Medium + TGFβ1 (PM+T). The positive medium consisted of DMEM-HG with l-Glutamine (Gibco: 11965-092), 1% Penicillin/Streptomycin, 1% (v/v) ITS+ (Gibco: 41400-045), 1 mM Sodium Pyruvate (Sigma: S8636, 100 mL), 50 μg/mL l-Proline (Sigma: P5607, 25 g), 100 nM Dexamethasone (Sigma: D4902, 25 mg), and 150 μM ascorbic acid 2-phosphate (Sigma: 49752). Additionally, 10 ng/mL TGFβ1 (Peprotech) was added. Medium changes were carried out three times a week, while TGFβ1 and ascorbic acid 2-phosphate were added fresh upon each medium change.

#### 2.1.1 Biochemical Analysis

SPH were collected on day 14 for biochemical analysis, including sulfated GAG and DNA content. SPH were digested using 125 μg/mL papain in 0.1 M sodium acetate, 10 mM l-cysteine-HCl, and 50 mM EDTA (all from Sigma-Aldrich) at pH 6.0 and incubated at 55 °C under constant shaking for 18 h. The DNA content of each sample was quantified using the Quant-iT PicoGreen assay (Thermo Fisher, USA). The GAG content of each sample was measured using the dimethyl methylene blue dye-binding assay (DMMB, Blyscan, Biocolor Ltd., United Kingdom) and chondroitin sulfate as the standard. The GAG content of each SPH was normalized to the measured DNA content to calculate the GAG/DNA ratio. Here, 32 SPH were pooled as a single sample at each time point, and *n* = 3 for both assays.

#### 2.1.2 SPH Viability and Morphology

The viability of the SPH was determined using a Live/Dead assay (Invitrogen) using 0.2 μM calcein-AM (live stain) and 0.6 μM propidium iodide (dead stain) in serum-free medium for 60 min at 37 °C. Cell viability was monitored on days 0 and 14 using a confocal laser scanning microscope (LSM 800, Zeiss, Germany). The maximum Feret diameter of SPH was measured using images taken from an Echo Revolve 4K microscope (Echo, San Diego, CA, USA) on days 0 and 14.

#### 2.1.3 Gene Expression Analysis

RNA was extracted from SPH (24 SPH were pooled for each biological replicate) on days 0 and 14 after lysing with 0.5 mL of TRIzol Lysis Buffer (Sigma). The total RNA was extracted using the RNeasy Mini Kit (Qiagen, Netherlands), according to the manufacturer’s protocol. Subsequently, the RNA concentration was measured using a microplate reader, Synergy H1 (BioTek, USA). RNA concentration was diluted to result in 100 to 250 ng per reaction for the cDNA synthesis (reverse transcription) using the All-In-One 5× RT MasterMix (Applied Biological Materials, Canada). cDNA was diluted accordingly in nuclease-free water, and RT-qPCR was conducted using the primers in Table 1. For RT-qPCR, the following program was applied using the CFX Connect Real-Time PCR Detection System (Bio-Rad, USA): 3 min at 95 °C followed by 40 cycles of denaturation at 95 °C for 10 s and annealing/extension at 60 °C for 30 s. Gene expression was normalized to the housekeeping gene (RPLP0), while samples collected on day 0 (Expansion medium) were used to calculate relative gene expression using the double-delta Ct method.

**Table 1:** Primers Used for RT-qPCR Analysis

Gene	Gene name	Manufacturer, assay ID
*SOX9*	SRY (sex determining region Y)-box nine	Biorad—qHsaCID0021217
*ACAN*	Aggrecan	Biorad—qHsaCID0008122
*RPLP0*	Ribosomal protein, large, P0	Biorad—qHsaCEP0041375
*COL10A1*	Collagen type X, alpha 1	Biorad—qHsaCID0007356
*COL2A1*	Collagen type II, alpha one	Biorad—qHsaCED0001057
*KRT18*	Keratin 18	Biorad—qHsaCED0035037
*HIF1α*	Hypoxia-inducible factor 1, alpha subunit	Biorad—qHsaCID0014755
*IL6*	Interleukin 6	Biorad—qHsaCED0044677
*MMP13*	Matrix metallopeptidase 13	Biorad—qHsaCIP0026824

### 2.2 Optimization of 2PP Parameters for MS Production

We fabricated MS using our custom 2PP system and a commercially available PCL-based resin (DEGRAD INX, BIO INX, Belgium) as described in our previous studies.^16,28^ Briefly, the resin and 3 wt % photoinitiator OXE-01 2-((Benzoyloxy)­imino)-1-(4-(phenylthio)­phenyl)­noctan-1-one; abcr GmbH, Germany) were dissolved in Tetrahydrofuran (THF) at 50 °C for 2 h. After dissolution, the excess THF from the resin was evaporated at reduced pressure by maintaining a temperature of 50 °C. The photosensitive resin was transferred to sample holders, and printing was performed using a custom-built 515 nm femtosecond laser-based 3D printer with a resonant scanner. The optimal printing parameters were screened by varying the laser power (from 200 to 420 mW), line distance (dxy, from 0.4 to 1.7 μm), and layer distance (dZ, from 1.5 to 2.7 μm). After printing, the MS were developed using the previously mentioned method for PCL-based resins.^17^ Scanning electron microscopy (SEM) analysis was performed to identify the most suitable printing parameters that result in shape-accurate structures. For this, the MS were sputter-coated with Au and investigated by SEM (Evo 10, Zeiss). MS for cell seeding were surface-modified with heparin by following a previously published protocol.^28^ After this, the MS were deposited in 384-well plates (one MS per well, low-adherent plates prepared using Lipidure coating (Amsbio, USA) beforehand to prevent cells from attaching to the well).^29^ The MS-loaded well plates were sterilized under UV for 1 h inside a laminar flow and completely dried before cell seeding.

### 2.3 NP-like Differentiation of SPH and S-SPH

The initial expansion of hBMSC was performed, as outlined in Section 2.1. Once the cells reached 70–80% confluency, they were seeded in low-adhesion 384-well plates with or without MS (prepared as above) at a cell density of 2000 cells/SPH. After culturing the SPH and S-SPH in EM for 2 days, the samples were divided into four groups based on the composition of the medium used for further treatment. The first group was maintained in the expansion medium (EM) as described in Section 2.1. The second group was cultured in positive medium supplemented with TGFβ1, termed PM+T (also detailed in Section 2.1). The third group, referred to as PM+G, received positive medium with the addition of GDF5 (Peprotech-AF-120-01) at a concentration of 100 ng/mL. The fourth group, PM+T+G, was treated with positive medium containing both TGFβ1 (10 ng/mL) and GDF5 (100 ng/mL).

#### 2.3.1 SPH and S-SPH Viability, Morphology, Biochemical, and Gene Expression Analysis

Viability and morphology were assessed as described in Sections 2.1.1, 2.1.2, and 2.1.3. The COL2α1 and COL10α1 gene expressions were also quantified using the primers listed in Table 1.

#### 2.3.2 Mechanical Property Analysis

The apparent elastic modulus of individual SPH and S-SPH was measured on day 14 using a microscale parallel-plate compression testing system (Microtester) controlled by the SquisherJoy software (Microtester, CellScale, Canada). The SPH and S-SPH were placed in a PBS bath and compressed with the microbeams until they reached 50% of their diameter. The diameter of the microbeam was selected based on the compressibility of the spheroids (0.1014 and 0.3048 μm). In addition, the strain rate was maintained at 0.04/s, and the loading and unloading times were 12.5 s with 5 cycles. From the displacement and force data, the apparent elastic modulus was calculated using the Hertz formula up to 10% deformation, assuming uniform compression and treating the spheroid as a sphere.^28,30^

$$F=\frac{4}{3}R^{1/2}\cdot \frac{E}{1-\upsilon^{2}}\cdot d^{3/2}$$

Here, *F* represents the force (μN), *R* is the actual radius of the spheroids with or without microscaffold, *E* is the apparent Young’s modulus (Pa), υ is the Poisson ratio (assumed to be 0.5), and *d* is the displacement (μm).

### 2.4 Analysis of NP-like Differentiation under a Healthy *In Vivo*-like (Hypoxic and Low Glucose) Environment with S-SPH

A healthy IVD exhibits low glucose and oxygen concentrations.^31^ We were interested in evaluating the NP-like differentiation under these conditions as well. Therefore, the initial expansion of hBMSC was performed, as mentioned in Section 2.1. Once the cells reached 70–80% confluency, they were seeded in low-adhesion 384-well plates with MS at a cell density of 2000 cells/spheroid. The expansion medium was used for 2 days to culture the spheroids. After 2 days, the medium was changed into two different groups: (i) High glucose + Normoxia group (HG+NOR) consisting of Positive medium (following Section 2.1) + GDF5 and the culture condition was maintained at 21% O_2_ and (ii) Low glucose + Hypoxia group (LG+HYP) consisting of Positive medium (following Section 2.2 and GIBCO’s DMEM-LG without external sodium pyruvate) + GDF5 and the culture condition was maintained at 2% O_2_. Here, GDF5 was used at a concentration of 100 ng/mL.

#### 2.4.1 S-SPH Viability, Morphology, Mechanical, Biochemical, and Gene Expression Analysis

Sections 2.1.1, 2.1.2, 2.1.3, and 2.3.2 were followed for the experiments. Gene expression was measured for *KRT18* and *HIF1α* using the primers in Table 1.

#### 2.4.2 Histology and Immunohistochemistry of S-SPH

After 14 days, S-SPH from the LG+HYP group were fixed in Histofix (ROTI Histofix, P087.4) overnight at 4 °C and washed three times with PBS. Further, S-SPH were embedded in Tissue Tek O.C.T. (Sakura, Zoeterwoede, Netherlands) and frozen at −20 °C. Cryofixed molds were sectioned into 8-μm slices using a cryotome (Cryostar NX50, ThermoFisher) and then attached to a glass slide (SuperFrost Plus Gold, Epredia). The slides were stored at −20 °C until staining. The slides were stained using alcian blue staining solution (Sigma, TMS-010-C) for 1 h and were subsequently washed with PBS. Additionally, Picrosirius red staining solution (Morphisto: 13422) was used to stain the samples for 1 h. Subsequently the samples were washed once with 0.5% acetic acid (Sigma: 32009). All slides were dehydrated in a series of ethanol and mounted (Entellan new, Sigma) with a coverslip and imaged using LSM800 (Carl Zeiss, Germany).

For immunofluorescence of collagen type II, the slides were incubated with the blocking buffer (5% BSA + 0.1% Triton X-100 in PBS) for 1 h. The anticollagen II primary antibody (Rabbit, Abcam ab34712) was applied at a 1:100 dilution in blocking buffer and incubated overnight at 4 °C. After washing three times with PBS, the secondary antibody (Goat anti-Rabbit IgG (H+L) Cross-Adsorbed Secondary Antibody conjugated with Alexa Fluor 555, Thermo Fisher, A-21428) was applied at a concentration of 10 μg/mL for 1 h at room temperature in the dark. After three additional washing steps using PBS, the sections were mounted (ProLong Diamond Antifade Mountant with DAPI). For aggrecan staining, sections were blocked, followed by overnight incubation at 4 °C with the antiaggrecan antibody (Mouse, Abcam ab3778) at 0.05 μg/mL in blocking buffer. After washing, a secondary antibody (Goat anti-Mouse IgG (H+L) Superclonal Secondary Antibody, conjugated with Alexa Fluor 488, Thermo Fisher, A-28175) was used at a 1:2000 dilution for 1 h at room temperature in the dark. After being washed, the sections were mounted. Finally, the sections were imaged using an LSM800.

### 2.5 Analysis of Injectability Profile for SPH and S-SPH

The injection-related effects on morphology, cell viability, and stress-associated gene expression of nondifferentiated SPH and S-SPH were assessed. For this, hBMSCs were seeded in low-adhesion 384-well plates with or without MS at a cell density of 2000 cells/spheroid. After 2 days of culture, the spheroids were divided into 3 groups:

- Spheroids after 2 days of culture (before injection)
- Spheroids after injecting through a 26G needle in a 1 mL syringe 1 time
- Spheroids after injecting through a 26G needle in a 1 mL syringe 5 times

In all cases, the injection volume was fixed at 200 μL, and the test was performed 3 times. After injection, the spheroids were cultured for 24 h in a low-adherent 6-well plate to see the effect of the injection on the cell viability and stress-associated gene expression. The number of SPH or S-SPH for each injection was 500, reaching a total cell number of 1 × 10^6^.

The viability postinjection was measured using PrestoBlue cell viability reagent (Invitrogen, Fisher Scientific) according to the manufacturer’s protocol. SPH morphology assessment and Live/Dead assay were performed following Section 2.1.2. Moreover, RT-qPCR analysis of IL6 and MMP13 was performed as described in Section 2.1.3 using the primers listed in Table 1.

#### 2.5.1 Injectability Profile of NP-like S-SPH in an In Vitro Injection Model

A 2% (w/v) agarose solution was cast in a mold, and before gelation, an air bubble was formed with a syringe. The agarose solution was allowed to solidify at room temperature, leaving a cavity (air bubble) behind. Afterwards, the gel was immersed in medium for at least 24 h to equilibrate in the incubator. S-SPH were differentiated into NP-like using the protocol described in the previous section (Section 2.4). Following differentiation, 500 NP-like S-SPH were transferred into 200 μL of PBS and injected using a 26G needle into the cavity of the agarose gel. The whole construct was then cultured in EM for 2 days. Finally, the fused construct was stained for live/dead to estimate the viability.

### 2.6 Statistical Analysis

Statistical analyses were conducted using GraphPad Prism 8 (GraphPad Software, USA). Data normality was assessed with the Shapiro–Wilk test. For comparison, analysis of variance (ANOVA) followed by Tukey’s post hoc test (column analysis) was performed, with significance defined at *p* < 0.05. Results are expressed as mean ± standard deviation (SD), unless specified otherwise. Unless otherwise stated, *n* represents the number of independent biological replicates.

## 3 Results

### 3.1 Establishing the Seeding Density for MS

Cell therapy injection into the IVD is commonly performed using a 26G needle (inner diameter ∼ 260 μm);^32,33^ therefore, we were interested in producing MS with a diameter of 200 μm. To identify the cell seeding density for MS, we formed SPH with 2000 and 3000 cells and differentiated them in PM+T. Although SPH with 3000 cells exhibited a larger size on day 0 compared with those with 2000 cells, both groups reached similar dimensions by day 14 (Figure 2a,b). Despite the size reduction, qualitative analysis using Calcein AM (live cells) and propidium iodide (dead cells) staining confirmed that the SPH maintained a high cellular viability throughout the culture period (Figure 2c). To determine the optimal ECM production of SPH, we performed biochemical and gene expression analyses. Notably, after 14 days of differentiation, SPH seeded using 2000 cells produced similar GAG/DNA levels compared with those with 3000 cells (Figure 2d). Gene expression analysis revealed significant upregulation of key chondrogenic markers in the 2000 cells/SPH group. SOX-9 levels increased approximately 3-fold, and ACAN expression rose nearly 4-fold compared to that in the 3000 cells/SPH group (Figure 2e,f). Combined with the earlier observation of size convergence by day 14 (Figure 2a,b) and maintained viability (Figure 2c), these findings confirmed that 2000 cells/SPH provides the optimal balance of physical stability and differentiation capacity for chondrogenic development. The cell seeding density was fixed at 2000 cells/SPH for MS (=S-SPH) throughout the study.

**Figure 2:**
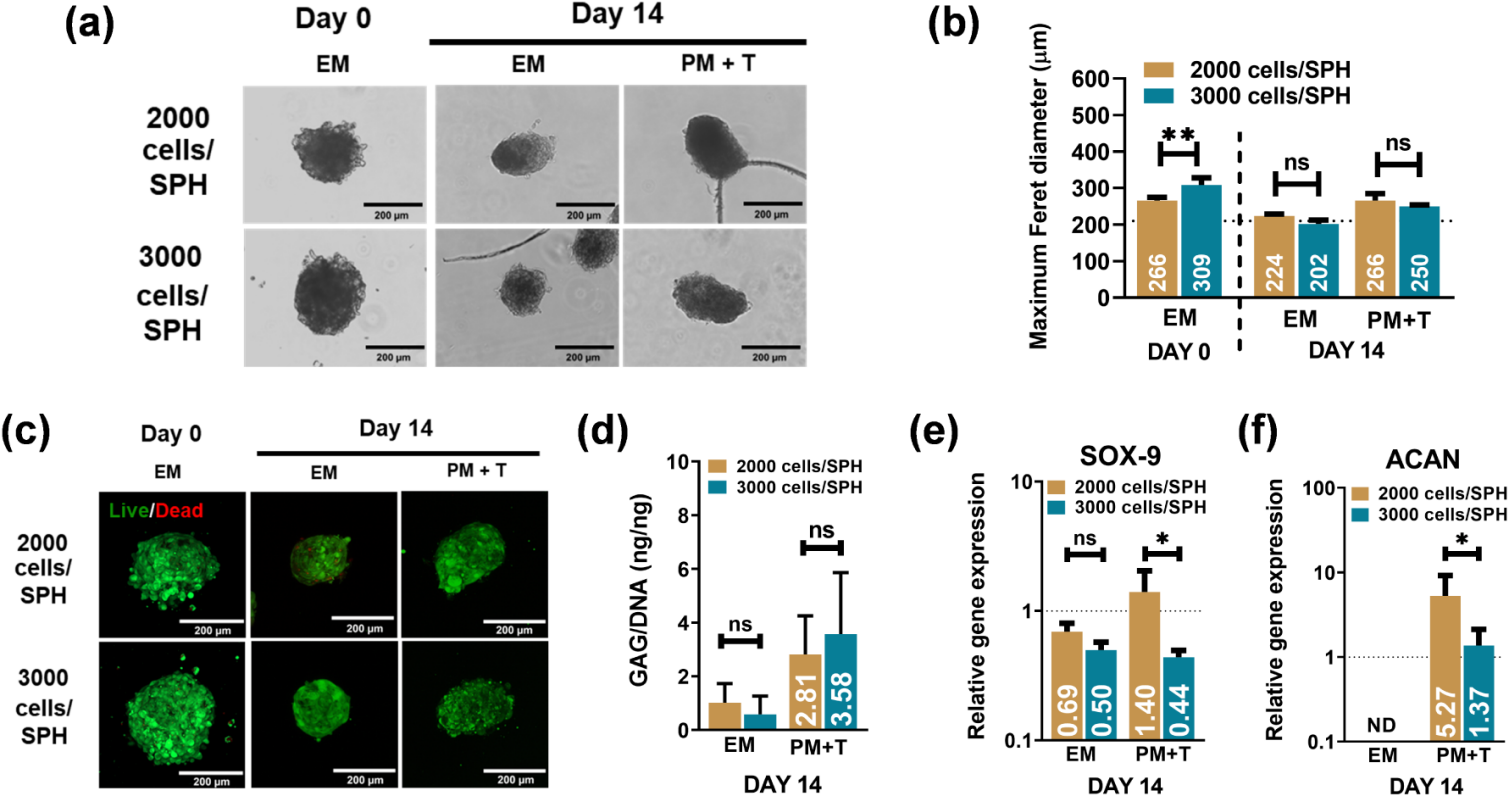
(a) Phase contrast images of SPH; (b) maximum Feret diameter of SPH with different seeding densities on days 0 and 14 (*n* = 3, biological replicates) (1 hBMSC donor); (c) live/dead images of SPH with different seeding densities on days 0 and 14; (d) ratio of GAG/DNA and gene expression profiles for (e) *SOX-9* and (f) *ACAN* (*n* = 3, biological replicates). Gene expression was normalized to the housekeeping gene (*RPLP0*), while samples collected on day 0 (Expansion medium) were used to calculate the relative gene expression. *, ** denote significance with *P* values <0.05 and <0.01, respectively.

### 3.2 Influence of GDF5 on the NP-like Differentiation of S-SPH

#### 3.2.1 Optimization of 2PP Printing Parameters for MS

We fabricated MS using a custom-built femtosecond laser-based (wavelength of 516 nm) 2PP system equipped with a resonant scanner,^16^ employing a PCL-based resin. Building on prior work from our group,^15^ we optimized critical printing parameters: laser power, line spacing, and layer spacing to ensure the structural stability of MS (Figure 3a). Our experiments showed that a minimum laser power of 200 mW was essential for producing stable MS. Further refinement of line and layer spacing (Figure S2a) revealed that a line spacing of 0.9 μm and a layer spacing of 2.3 μm resulted in the most stable and shape-accurate MS, as illustrated in the SEM image (Figure S2d). Representative SEM images of the MS (printable and not printable, Figure S2b,c), including comparative results, are shown in Figure S2a–d, confirming the efficacy of the optimized parameters.

**Figure 3:**
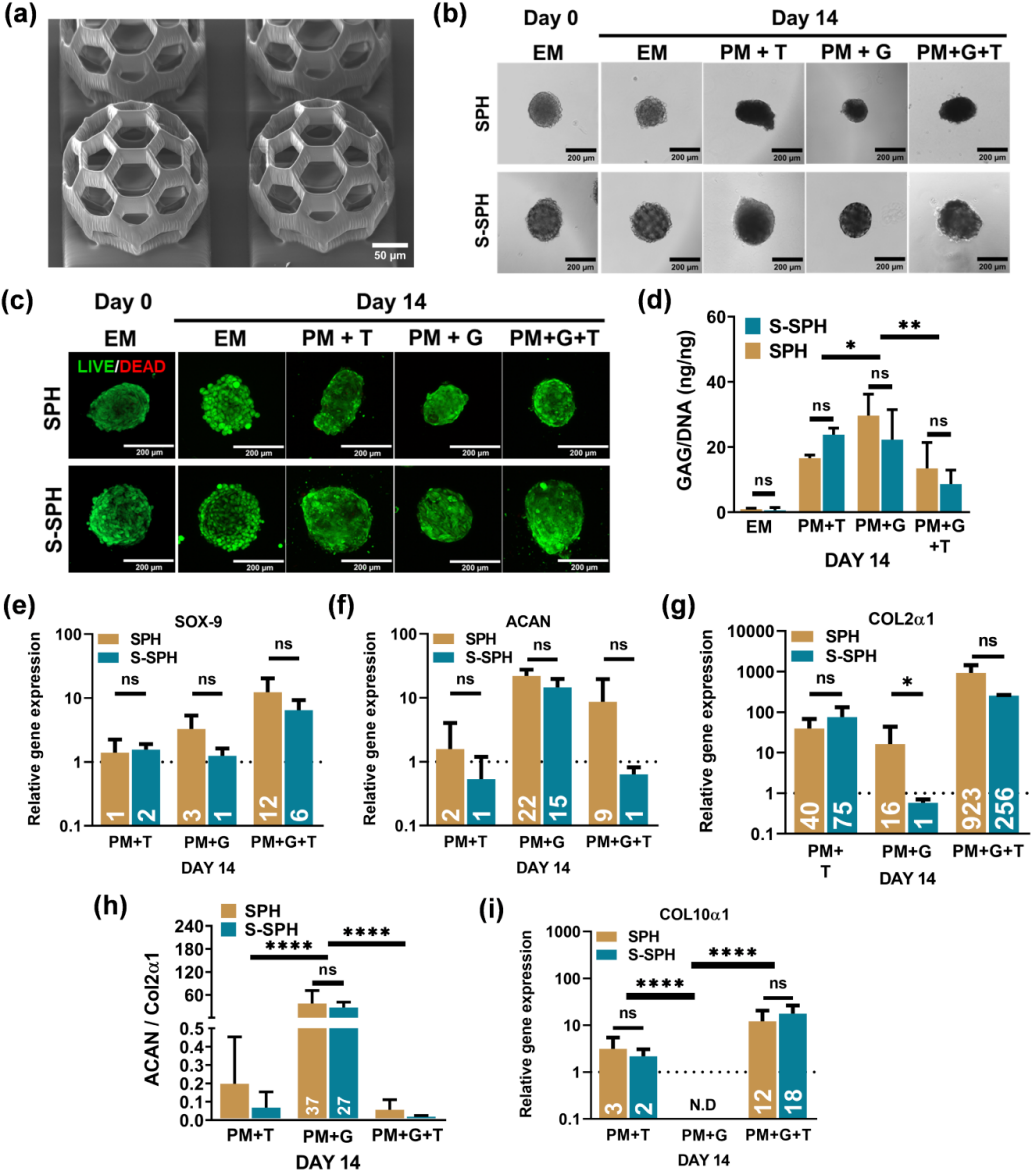
(a) SEM image of MS; EM means Expansion medium, PM+T means Positive medium + TGFβ1, PM+G means Positive medium + GDF5, and PM+G+T means Positive medium + GDF5 + TGFβ1; (b) phase contrast and (c) live/dead images of SPH and S-SPH on days 0 and 14; (d) GAG/DNA ratio, relative gene expressions of (e) *SOX-9*, (f) *ACAN*, (g) *COL2α1*, (h) *ACAN/COL2α1*, and (i) *COL10α1* for SPH and S-SPH on day 14 (*n* = 3, biological replicates) (1 hBMSC donor). *, **, **** denote significance with *P* values <0.05, <0.01, and <0.001, respectively.

#### 3.2.2 Analysis of Morphology and Biochemical Composition of SPH and S-SPH

Based on previous literature, TGFβ1 (10 ng/mL), GDF5 (100 ng/mL), and the combination of both were selected as growth factors promoting NP-like differentiation in the hBMSC spheroids. MS accommodated SPH formation and retained the viability and shape. We cultured S-SPH with 2000 cells per construct, yielding a diameter of 213 μm (Figure S3), which were subsequently differentiated for 14 days. Phase-contrast imaging (Figure 3b) highlighted distinct morphological changes: TGFβ1 supplementation induced elongation and irregularity in SPH morphology, while GDF5 treatment reduced the spheroid size. Notably, S-SPH under GDF5 supplementation retained the original dimensions and roundness of the MS template. Importantly, none of the growth factors had an adverse effect on cellular viability, as confirmed by the high viability observed in both SPH and S-SPH on day 14 (Figure 3c). Further, we quantified GAG production (normalized to DNA content) after 14 days of differentiation (Figure 3d). We observed no significant differences in GAG/DNA ratios between SPH and S-SPH, regardless of growth factor supplementation. Notably, supplementation of GDF5 alone resulted in the highest GAG/DNA levels in both groups (SPH and S-SPH), whereas TGFβ1 and the TGFβ1/GDF5 combination showed lower values, with the lowest GAG/DNA recorded in the combinatory group.

#### 3.2.3 Analysis of Gene Expression of SPH and S-SPH

We further analyzed chondrogenic markers (*SOX9*, *ACAN*, *COL2α1*) and the hypertrophy-associated gene *COL10α1*. While gene expression levels were largely comparable between SPH and S-SPH across all conditions, distinct trends emerged based on growth factor supplementation. *SOX9* expression was relatively suppressed in groups treated with TGFβ1 or GDF5 alone, while it showed an upward trend in the combinatory group (Figure 3e). *ACAN* expression was the highest in the GDF5-treated group, whereas TGFβ1 and the combination group exhibited lower levels (Figure 3f). *COL2α1* expression was the highest in the combinatory group, followed by the TGFβ1 group (Figure 3g). Strikingly, the *ACAN/COL2α1* ratio, a marker of balanced chondrogenesis and the NP phenotype, was most elevated in the GDF5 group, diminishing significantly under TGFβ1 or combinatory conditions (Figure 3h). Finally, *COL10α1*, a hypertrophic marker, was undetectable in the GDF5 group but showed modest expression in the TGFβ1 and combinatory group (Figure 3i). These findings, summarized in Figure 3d–i, underscore GDF5′s capacity to enhance ACAN-rich ECM while suppressing hypertrophy, positioning it as a candidate for NP-like differentiation of S-SPH. Based on these observations, GDF5 was selected to induce differentiation in the remaining study.

### 3.3 Effect of MS on the Mechanical Properties of S-SPH

To evaluate the mechanical properties of S-SPH and SPH, we performed cyclic compression testing using a CellScale microtester. The cyclic compression test protocol was adapted from previous studies, which have been used to analyze intact IVD with 5 cycles.^34^ We replicated it in our MS with slight modifications using the microtester. After analysis, MS maintained a similar displacement–force curve throughout 5 cycles, as depicted in Figure 4a. The reduction in the maximum force after each cycle was less than 5%, compared to the force at the maximum displacement after the first cycle. The shape of MS was maintained even after 5 cycles, which confirmed the highly elastic nature of MS (Figure 4a). In contrast, SPH differentiated for 14 days under GDF5 supplementation showed progressive deterioration of mechanical properties, losing ∼30% of the maximum force after 5 cycles of loading (Figure 4b). However, culturing spheroids within MS (S-SPH) significantly mitigated this decline, reducing the maximum force loss to ∼15% (Figure 4c). Digital images (Figure 4d) further illustrated the preserved integrity of S-SPH postcompression compared to disintegrated SPH. As a result, the S-SPH group resulted in an average maximum force of 498 μN (Figure 4e) compared to SPH, which reached only 81 μN, a value that is 6 times lower. Ultimately, SPH’s apparent Young’s modulus (*E*) was 18 kPa, while for S-SPH it increased to 91 kPa.

**Figure 4:**
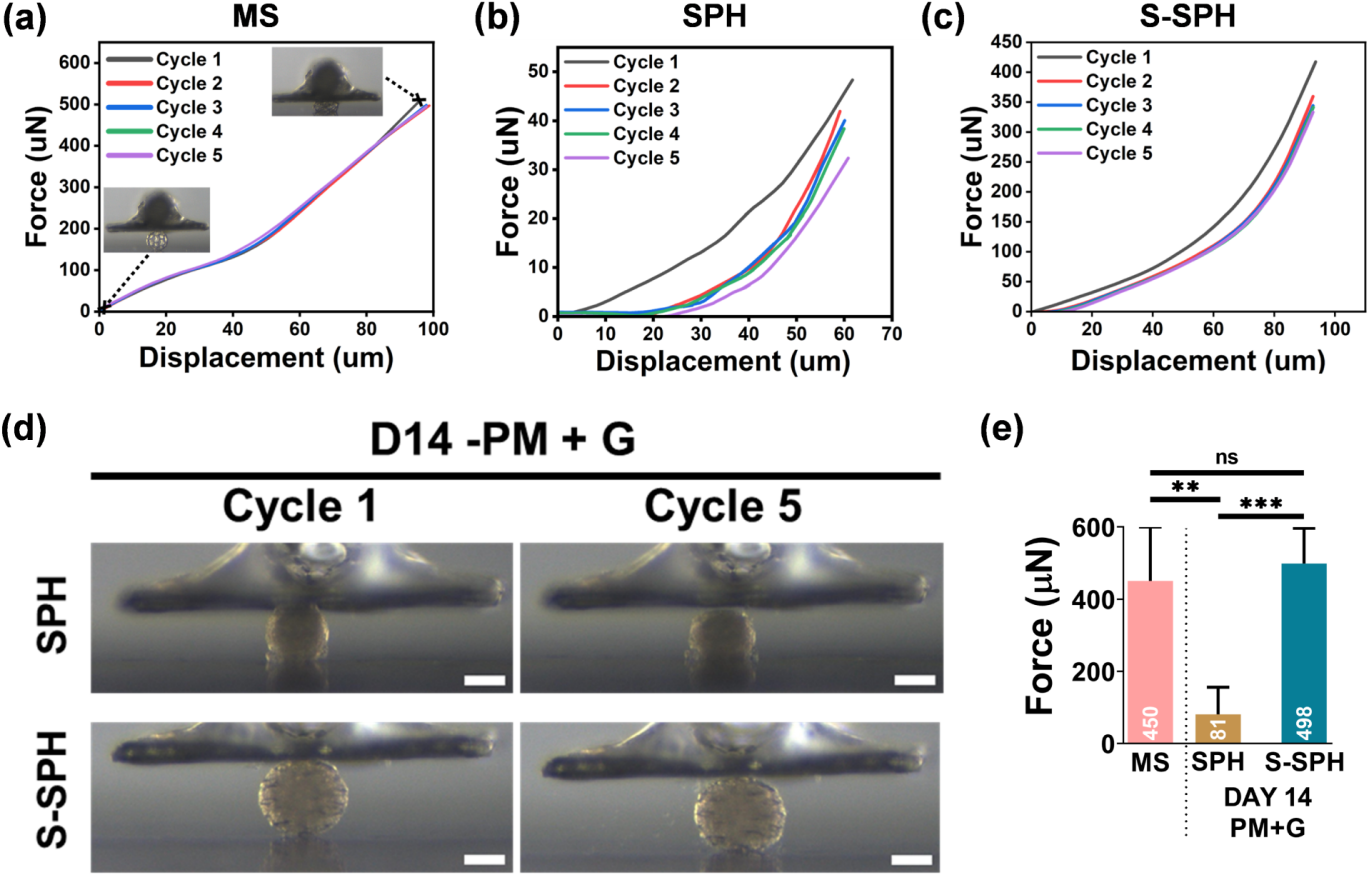
Cyclic compression test results of (a) MS, (b) SPH, and (c) S-SPH, which were cultured for 14 days in PM+G; (d) images of SPH and S-SPH, which were cultured for 14 days in PM+G, during the cyclic compression test. The images represent the initial shape before the first cycle and after the fifth cycle; (e) comparison of maximum force between MS, SPH, and S-SPH (*n* ≥ 3, biological replicates). **, *** denote significance with *P* values <0.01 and <0.001, respectively.

### 3.4 Healthy *In Vivo*-like (Hypoxia and Low Glucose) Environment-Mediated Changes in the NP-like Phenotype and Mechanical Properties of S-SPH

To assess whether a physiologically relevant, healthy *in vivo* (NP-mimetic) environment enhances the differentiation potential of S-SPH, we cultured S-SPH under hypoxic conditions (2% w/v O_2_) with low glucose (LG+HYP). GDF5 supplementation was compared to standard high glucose, normoxic conditions (HG+NOR). After 14 days, S-SPH cultured in LG+HYP retained their original size and spherical morphology (Figure 5a), confirming a sustained high cell viability (Figure 5a). Quantitatively, the LG+HYP group exhibited a 2-fold increase in the GAG/DNA ratio compared to HG+NOR (Figure 5b), without significant differences in the amount of DNA between the two groups, underscoring ECM synthesis under NP-like conditions. Hypoxia-driven *HIF1α* expression resulted in a several 1000-fold increase in LG+HYP compared to that under HG+NOR conditions (Figure 5c), aligning with the hypoxic niche of native NP tissue. Additionally, *KRT18*, an important NP phenotypic marker, was upregulated ∼19-fold in LG+HYP relative to HG+NOR (Figure 5d). Similarly, the level of *ACAN* expression increased significantly (96-fold, Figure 5e), further corroborating NP-like differentiation along with ECM maturation. The mechanical properties of S-SPH cultured in LG+HYP conditions increased, as determined by cyclic compression tests. The maximum force (at maximum displacement of the first cycle) and apparent elastic modulus were increased upon stimulation under LG+HYP to 1643 μN and 217 kPa, respectively, compared to 498 μN and 91 kPa under HG+NOR (Figure 5f,g). The increased elastic modulus correlates well with the native human IVD modulus reported previously in the range from 160 to 300 kPa.^35,36^ Additionally, the effect of LG+HYP and GDF5 on S-SPH was analyzed by histology, and representative images are shown in Figure 5h_1_–h_4_. Alcian blue staining was uniform and intense throughout the S-SPH, and no zonal unevenness was observed (Figure 5h_1_). The aggrecan staining was prominent, which confirmed that the specific NP-ECM was secreted and maintained through the differentiation process. Furthermore, a network-like distribution of aggrecan was identified throughout the S-SPH section (Figure 5h_2_). Moreover, picrosirius red staining was positive in the S-SPH group, and tissue-specific collagen type II secretion was further confirmed by IHC staining (Figure 5h_3_–h_4_).

**Figure 5:**
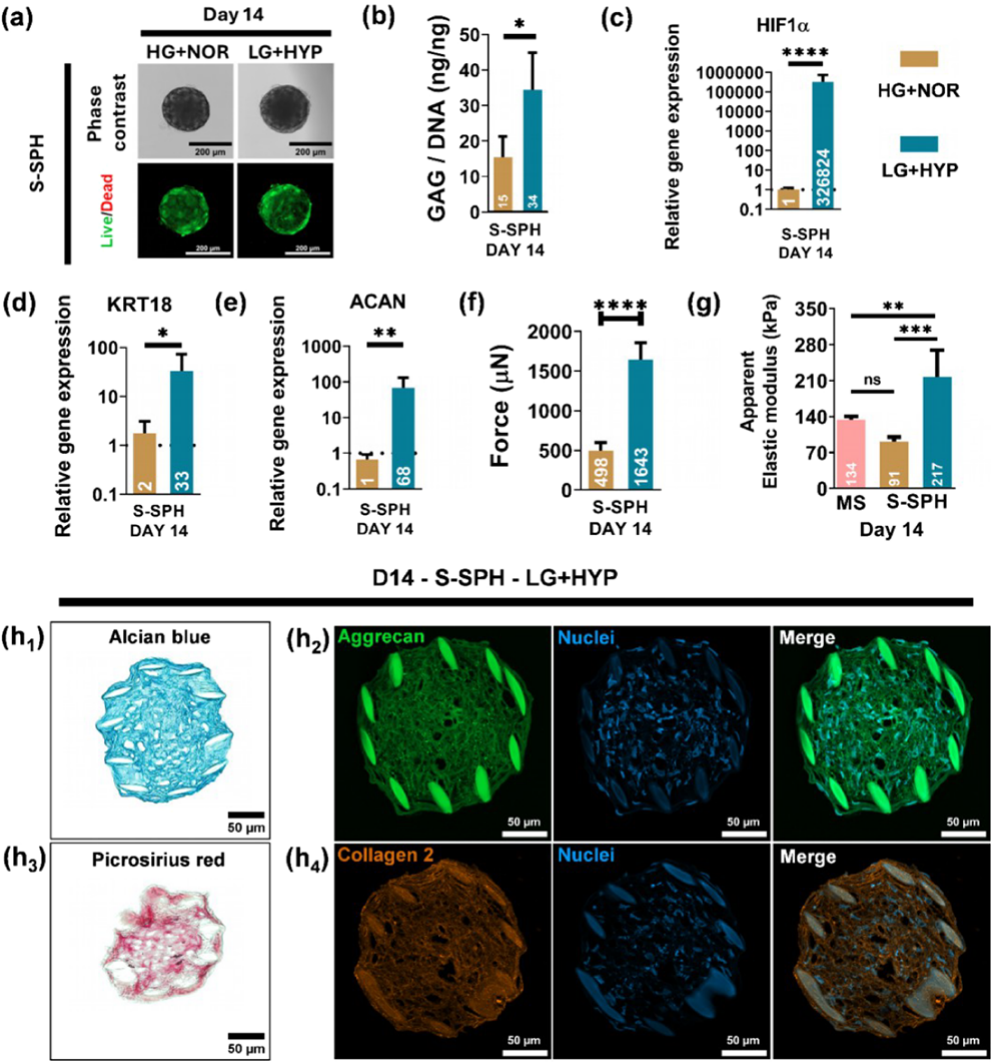
(a) Phase contrast and live/dead images of S-SPH on day 14; (b) GAG/DNA ratio and relative gene expression of the genes (c) *HIF1α*, (d) *KRT18*, and (e) *ACAN* of S-SPH normalized to the housekeeping gene *RPLP0*, while samples collected on day 14 (HG+NOR) were used to calculate the relative gene expression; (f) maximum force at the first cycle and (g) apparent elastic modulus for S-SPH assessed through cyclic compression tests; (h_1_) Alcian blue, (h_2_) Aggrecan, (h_3_) Picrosirius red, and (h_4_) Collagen 2 staining for S-SPH. *, **, ***, **** denote significance with *P* values <0.05, < 0.01, < 0.001, and <0.0001, respectively (*n* = 3, biological replicates) (1 hBMSC donor).

### 3.5 Effect of Injection Procedure on the Viability of Cells in SPH and S-SPH

We tested the effect of injection in SPH and S-SPH by delivering 500 units through a 26G needle using 200 μL of PBS as a carrier (Figure 6a–e). We compared the effects of 1 and 5 repeated injections on morphology, viability, and stress-associated gene expression. Phase-contrast images revealed that the sizes of SPH and S-SPH remained largely unaltered after the first injection (Figure 6a). However, following the fifth injection, a noticeable reduction in SPH size was observed, whereas S-SPH maintained its original dimensions (Figure SI4). Viability was assessed by live–dead staining and with metabolic activity measurements (Figure 6b and c). Results indicated that the cells in both SPH and S-SPH retained a high viability after the first injection. After the fifth injection, the cells in SPH exhibited a significant decrease in viability compared to the preinjection level. In contrast, the cells in S-SPH remained viable, with no significant changes compared to preinjection values. Stress-related gene expression profiles revealed critical differences between the two groups. After the first injection, *IL6* (a pro-inflammatory marker linked to mechanical damage) expression decreased in both groups (remained higher in SPH) (Figure 6d). Following the fifth injection, S-SPH exhibited ∼16-fold lower *IL6* expression than did SPH (Figure 6d). MMP13, a matrix-degrading enzyme, increased 1.8-fold in SPH after the first injection, while S-SPH showed no rise (Figure 6e). After the fifth injection, *MMP13* expression surged in SPH but was 4.7-fold lower in S-SPH (Figure 6e). The higher expression of *IL6* and *MMP13* in SPH after the fifth injection clearly indicates that the cells undergo a catabolic phase, which is reduced to a certain extent in S-SPH. After confirming the injectability profile of undifferentiated S-SPH, we analyzed how the NP-like S-SPH would be affected by the injection/application in an *in vitro* injection model (Figure 6f). In this experiment, we found that the injectability was effective for the NP-like S-SPH that fused/agglomerated to form a larger tissue construct with minimal cell death after injection/application. (Figure 6g).

**Figure 6:**
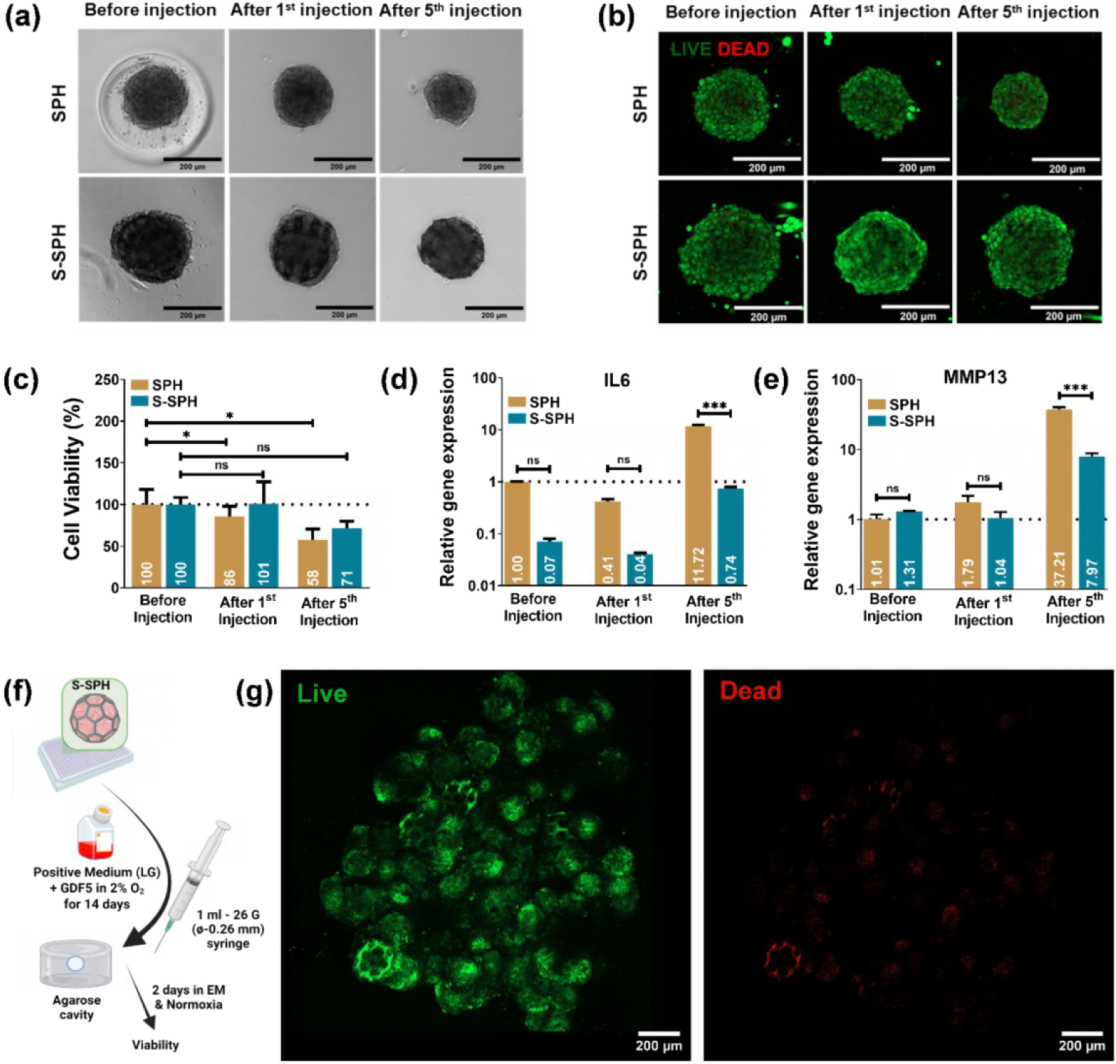
Assessment of injectability profile on SPH and S-SPH; (a) phase contrast and (b) live/dead images; (c) cell viability and relative gene expressions for (d) *IL6* and (e) *MMP13*. Gene expression was normalized to the housekeeping gene (*RPLP0*), and relative gene expression was calculated compared to samples taken before the injection. (f) Schematic of the work plan to inject NP-like spheroid in the *in vitro* injection model (Created in BioRender. Balasubramanian (2026) https://BioRender.com/b3urgom) (g) Live/dead images of fused S-SPH after 2 days of injection into the injection model. *, *** denote significance with *P* values of <0.05 and <0.001, respectively, *n* = 3, biological replicates (1 hBMSC donor).

## 4 Discussion

A minimally invasive therapy for IVD repair requires injectable tissue-building blocks that can withstand the harsh mechanical environment of the spine while delivering NP-regenerative cells. Building on our prior work with S-SPH for osteochondral repair,^18^ we adapted this strategy as a proof-of-concept platform for NP-targeted delivery. In our previous study, we demonstrated that highly porous MS could support the formation of hASC spheroids without impairing cell viability or differentiation potential.^18^ Here, we adapted and optimized the MS design for IVD application by downsizing the MS to be compatible with delivery/application through a thin needle. Importantly, the S-SPH retained the capacity for NP-like differentiation, indicating that the MS’s presence did not hinder the cells’ maturation and differentiation toward an NP phenotype. This S-SPH platform yields NP-primed tissue-building blocks with potential for minimally invasive disc delivery and provides a delivery-enabling strategy, rather than a comprehensive model of disc regeneration.

Recent literature about injection-related damage to IVD suggested that the needle diameter should be in the range of 8–15% of the disc height.^37^ It was reported that using a 21G needle injection caused more damage than a 30G needle in the rat IVD.^38^ Another study elucidated the risk of leakage from the disc based on the needle diameter, and the authors proposed a correlation between the two.^39^ Based on these previous studies, we chose 26G needles (inner diameter ∼260 μm) to deliver a volume of 200 μL. To align with this needle specification, we fabricated the MS with a diameter of 200 μm using 2PP and optimized hBMSC seeding densities and chondrogenic differentiation media, based on data from the previous work,^15^ to prevent oversize S-SPH formation postdifferentiation. After 14 days, SPH with 2000 cells maintained their size and exhibited substantial ECM production. Notably, these SPH showed higher expression levels of *SOX9* and *ACAN* compared to SPH containing 3000 cells/SPH. Our observations are consistent with the findings of Sarem et al., who reported that lower cell numbers in SPH enhance cell-to-cell interactions and improve the diffusion of supplements, leading to increased expression of N-cadherin and, consequently, *SOX9*.^40^ Another study proposed that microtissues with a low cell number had better oxygen penetration, which could increase the production of GAG and collagen, highlighting the importance of cell seeding density for microtissues.^41^ We propose that the improved diffusion in spheroids with lower cell densities contributed to the preservation of a larger population of healthy, matrix-producing cells, resulting in elevated *SOX9* and *ACAN* levels.

The advantage of 2PP over other 3D printing technologies is that it allows the fabrication of highly delicate structures such as MS, with struts as thin as 20 μm, through the high achievable spatial resolution of this technology. Recent advancements in 2PP technology, made within the research group, have significantly enhanced the fabrication throughput.^16^ Specifically, the integration of the resonant scanner technology into our 2PP system has led to a 10-fold fabrication speed compared to our previous setup.^15^ The laser power, line, and layer spacing were varied to determine the printability region. The final printing parameters were chosen based on the shape accuracy of the printed structures compared to the CAD file. After optimizing the printing parameters, the MS were produced at a speed of 66 m/s. In terms of material degradation properties, we have previously demonstrated the degradability of the material used to fabricate the MS presented here (UPCL-6).^17^ In an accelerated degradation study, the degradation rate of UPCL-6 was similar to that of commercial linear, non-cross-linked PCL (10,000 g mol^–1^). These data may indicate that complete degradation once implanted would take several years, as reported for non-cross-linked PCL.^42^ The degradation occurs through hydrolytic degradation, involving the random scission of ester bonds. The slow degradation of UPCL-6 could avoid the local increase in acidity which occurs upon degradation of other materials, such as polylactic acid (PLA) or poly­(lactic-co-glycolic acid) (PLGA), which degrade faster.^43^ Unpublished work from our laboratory has shown good results using differentiated S-SPH for osteochondral defect repairs in a rabbit *in vivo* model. Microscaffolds were still evident in the implantation site after 3 months, as expected. No signs of sustained inflammation were detected, and good integration between the implant and the surrounding host tissue was observed.

SPH and S-SPH were formed, and the analysis of NP-like differentiation was performed. TGFβ1 has been previously explored as a growth factor that helps hBMSC differentiation toward cartilage-like phenotypes. Additionally, we have used GDF5 which has shown promising results with large pellets of hBMSC,^44–46^ NP cells, and nasal chondrocytes^46,47^ to induce the discogenic lineage. Compaction and irregular shape over 14 days were avoided to a certain extent in the presence of MS. A similar observation was mentioned in our previous studies where S-SPH compacted less compared to SPH.^15^ The high viability of cells in SPH and S-SPH was confirmed via Live/Dead staining. GDF5 alone helped to produce GAG contents comparable to those of TGFβ1 and the combination of both, confirming its efficiency. The literature supports this finding showing that the GAG production difference was nonsignificant when hBMSC were differentiated with either TGFβ1 or GDF5.^48^ The combination of TGFβ1 and GDF5 was not as prominent in inducing differentiation into an NP-like lineage, which may be due to receptor competition or feedback inhibition, and warrants further investigation. Notably, GDF5 led to a marked upregulation of *ACAN* expression and an increased *ACAN/COL2α1* ratio in both SPH and S-SPH. Meanwhile, *COL2α1* and *COL10α1* expression increased in the presence of TGFβ1. The differing effects of these growth factors can be attributed to their distinct signaling pathways: GDF5 primarily signals through BMPRII and the Smad 2/3 pathway, associated with NP-like differentiation and ECM synthesis,^49^ while TGFβ1 signals through the Smad 1/5/8 pathway, which is linked to hypertrophic differentiation.^1^ Our results suggest that under these specific culture conditions, this differential signaling may favor GDF5 for driving NP-like differentiation and GAG production while minimizing hypertrophic marker expression. Importantly, the *ACAN/COL2α1* ratio exceeded 20 in the GDF5-treated group, a key indicator of the NP-like phenotype^50^ observed in both SPH and S-SPH. Based on these outcomes, GDF5 was utilized in subsequent steps for inducing NP-like differentiation in hBMSC spheroids (SPH and S-SPH) due to its effectiveness in promoting relevant gene expression and matrix production without inducing hypertrophy in this model.

Previous studies from our group have explained the compressive property of bulk material of UPCL6 ^17^ as well as MS (300 μm in diameter).^28^ The current study provides in-depth information about the mechanical properties of MS, which can be an important aspect of using MS to support load-bearing applications. After 14 days of differentiation with GDF5, S-SPH showed less deformation, a higher maximum force (necessary for compression), and an increased apparent elastic modulus compared to SPH, likely due to the MS acting as a reinforcing “exoskeleton” that prevents compaction and maintains shape, highlighting its potential as a mechanically robust scaffold. Altogether, the NP-like S-SPH was chosen as a suitable microtissue displaying good mechanical properties.

We analyzed the differentiation and mechanical properties of S-SPH in a hypoxic and low-glucose environment, mimicking the healthy *in vivo* environment. Synergistic anabolic signals, such as low glucose and hypoxia, along with GDF5, produced higher GAG/DNA ratios than culture in high glucose and normoxia. Literature suggests that under hypoxic conditions, hBMSC tend to have higher expression of *HIF-1α* and Notch which further upregulate the *ACAN* and *COL2α1* genes.^51,52^ This effect has previously been proven to be amplified by the presence of GDF5.^48^ At the same time, the absence of high-glucose levels resulted in fewer catabolic processes, such as matrix degradation via Matrix Metalloproteinases/A Disintegrin And Metalloproteinase with Thrombospondin Motifs (MMP/ADAMTS).^48^ The net effect is a pro-anabolic balance, so proteoglycans accumulate more under the low-glucose and hypoxia condition. Experiments have shown that hypoxia alone can increase GAG production of NP cells and hBMSC,^48^ and with GDF5, this GAG production and *ACAN* expression is enhanced. Thus, the healthy *in vivo*-like environment provides an ideal niche for matrix assembly. Studies have confirmed that *KRT18* is highly expressed in young, healthy NP cells but not in degenerated discs or other cartilage tissues.^53^ In our study, we observed strong upregulation of *KRT18* confirming that the S-SPH acquired a molecular signature of native NP cells. This is likely due to the combined effect of hypoxia, low-glucose levels, and GDF5 supplementation. GDF5 has been shown to decelerate IVDD progression and to promote the expression of healthy NP cell markers.^54,55^ Therefore, the healthy *in vivo*-like culture conditions not only increased general chondrogenic genes but also pushed S-SPH specifically toward an NP lineage fate. This was confirmed by both histology analysis and immunostaining of aggrecan and collagen type 2. Several studies reported that the healthy IVD elastic modulus was between 160 and 300 kPa,^35,36^ and this mechanical property was only achieved in the S-SPH group under healthy *in vivo*-like environment. Altogether, the healthy *in vivo*-like environment enhanced S-SPH differentiation and mechanical properties by promoting an anabolic balance. These conditions may be optimal for priming the S-SPH before injection into the degenerated disc environment. The effect of priming is planned to be evaluated *in vitro* in the future, by culturing in degenerated disc conditions, such as inflammatory cues, acidic pH, hyperosmolarity, and chronic mechanical overload, and *ex vivo* (10.1002/btm2.70120) in a degeneration model.^56^

Injecting cells through a small-diameter needle generates forces that can tear cell membranes apart, leading to cell death.^57^ In addition to the one-time injection, we conducted repeated injections to validate the injectability. The methodology for using repeated injections shows the robustness of using MS to protect cells even under exaggerated conditions. This approach enabled us to validate the delayed response of inflammation and catabolism that occurs through repeated injections. We found that S-SPH showed a higher viability after the first injection compared to SPH. The cell survival in SPH after the first injection may be prominent, as it allows for the delivery of SPH without cell death, since many cells typically die off due to the injection.^57^ Cytokines like IL6 cause inflammation of the disc and may lead to further complications, activation of catabolic pathways, as well as pain.^58^ Lower expression of IL6 in S-SPH may create a less inflammatory environment after injection, potentially improving long-term outcomes.^13^ MMP13 is a catabolic enzyme which breaks down the IVD’s ECM, leading to IVDD.^59^ In our study, *MMP13* expression was lower in S-SPH after the first injection, which suggests that MS can do both: protect cells and help to reduce the expression of catabolic factors. To quantitatively validate the physical integrity of S-SPH, we analyzed the Feret diameter before and after injection. The S-SPH exhibited no significant changes in the Feret diameter, maintaining their structural fidelity after one injection. Furthermore, we observed a nearly 100% S-SPH recovery rate with no incidence of needle clogging, confirming the suitability of the 26G needle size. In the context of repeated injections, S-SPH presented higher viability, lower *IL6* and *MMP13* expression, further supporting the role of the microscaffold as a protective carrier rather than merely a structural additive. Further analysis of S-SPH (NP-like) injection in an *in vitro* injection model revealed that good injectability was maintained after differentiation and that individual S-SPH tended to fuse upon injection rather than remain as discrete units. Although the S-SPH fused to form a larger tissue, the integration into the surrounding NP tissue needs to be evaluated through an *ex vivo* study in the future.

Many approaches have been proposed to tackle IVD regeneration,^11^ including the injection of single-cell suspensions, the use of acellular or cell-laden hydrogels, as well as the injection of cell spheroids. While several hydrogel-based approaches have been explored, they frequently lack the required mechanical stability for intradiscal injection.^60^ Alternatively, microscaffolds fabricated via electrospinning using poly­(lactic-co-glycolic acid) (PLGA) and femtosecond laser structuring have been utilized to support human NP cells.^61^ However, the degradation products of PLGA can decrease the local pH, which is detrimental to regeneration. Furthermore, the PLGA microscaffolds reported in the literature were highly populated but suffered from cell distribution confined to a single plane. Recent studies show that increased cell–cell contacts in 3D spheroids promote enhanced ECM deposition protecting cells in the harsh environment of the degenerated disc, and have increased retention upon injection, compared to single-cell suspension approaches.^62^ To promote the differentiation of ADSCs into NP-like cells, researchers seeded ADSCs onto microcarriers based on decellularized NP spheroids.^63^ While highly bioactive, decellularized microcarriers are not mechanically robust to withstand the loading of the spine. The strategy followed in our study aims at combining the benefits of cell clustering (cell spheroids *vs* single cells) and the increased mechanical stability provided by the MS, tackling current limitations of other approaches.

The current study focuses on IVD regeneration as the intended application; therefore, it was designed as a proof-of-concept focused on injectable delivery, mechanical protection during administration, and early maintenance of an NP-like phenotype. Accordingly, our experimental design incorporated key healthy NP-mimetic conditions (hypoxia and low glucose) for improved differentiation and injectability. Future studies will evaluate the use of differentiated S-SPH under the complex and harsh environment of a degenerative disc, including acidic pH, hyperosmolarity, chronic mechanical overload, and the presence of inflammatory cytokines. The experiments conducted here employed hBMSC from a single donor. Although biological replications were performed, this limits the overall conclusion regarding the effect of growth factor supplementation and the observed gene expression responses, as donor-to-donor variability is a known factor influencing hBMSC behavior. Future work will aim to validate these findings across a donor pool to ensure their clinical applicability.

## 5 Conclusion

In summary, we developed an S-SPH platform by seeding 2000 cells that preserved the spheroid morphology and high cell viability. These S-SPH remained structurally intact throughout the differentiation culture, underscoring the compatibility of MS with spheroid formation. Moreover, among the differentiation cues, GDF5 was the most potent factor for driving NP-like differentiation in S-SPH. GDF5-treated S-SPH expressed significantly elevated levels of *ACAN* with a high *ACAN/COL2A1* ratio and no *COL10A1* expression, indicating an NP-like phenotype without hypertrophic differentiation. S-SPH exhibited a higher elastic modulus and greater resistance under cyclic loading than SPH, reflecting the MS reinforcement effect. Notably, under healthy *in vivo*-like culture conditions (hypoxia and low glucose), S-SPH further upregulated key discogenic markers (*KRT18*, *ACAN*) and deposited more GAG/DNA, resulting in substantially increased mechanical properties close to those of human IVDs. Crucially, cells in the S-SPH demonstrated higher viability after injection and only minimal expression of inflammatory (*IL6*) and catabolic (*MMP13*) genes. In contrast to conventional cell therapy that often experiences cell loss and damage during needle injection, the S-SPH showed negligible adverse effects, highlighting the protective role of the MS during injection. Altogether, this study establishes a proof-of-concept injectable S-SPH platform that enhances cellular delivery and preserves early NP-like phenotypes. Future studies will evaluate this potential therapy in *ex vivo* IVDD models.

## Supplementary Material



## Data Availability

The data supporting the findings of this study are available from the corresponding author upon reasonable request, subject to availability.

## Notes

Trilineage analysis: GAG/DNA quantification, Alizarin red staining, and Bodipy lipid staining, after 18 days of differentiation in chondrogenic, osteogenic, and adipogenic medium, respectively (Figure S1); printability map of 2PP printing parameters, and corresponding SEM images (Figure S2); quantification of Feret diameter during chondrogenic differentiation culture in MS (Figure S3); and Feret diameter before and after injection of SPH and S-SPH (Figure S4) (PDF)
