# Supplementary material for: Exploring Injectable Scaffolded Spheroids for Nucleus Pulposus Therapy in Degenerated Intervertebral Discs
Source: ACS Appl Mater Interfaces. 2026 Feb 16;18(7):10994–1007. doi: 10.1021/acsami.5c24306 (PMC12954655; doi:10.1021/acsami.5c24306)
Supplement: Supplementary file 1 [file am5c24306_si_001.pdf]

Supporting Information

Exploring Injectable Scaffolded Spheroids for Nucleus Pulposus Therapy in Degenerated Intervertebral Discs

Rathina Vel Balasubramanian[1,2], Marcia Muerner[3,4], Oliver Kopinski-Grünwald[1,2], Sibylle Grad[3,4], Julia Fernández-Pérez[1,2,* #], Aleksandr Ovsianikov[1,2,* #]

[1]3D Printing and Biofabrication Group, Institute of Materials Science and Technology, Technische Universität Wien, Vienna 1040, Austria.

[2]Austrian Cluster for Tissue Regeneration (https://www.tissue-regeneration.at)

[3]AO Research Institute Davos, Clavadelerstrasse 8, Davos 7270, Switzerland

[4]Federal Institute of Technology Zurich, Zurich 8092, Switzerland

* Corresponding authors:

Julia Fernández-Pérez

Email: julia.fernandez.perez@tuwien.ac.at

Aleksandr Ovsianikov

Email: aleksandr.ovsianikov@tuwien.ac.at

[#] Authors contributed equally

1) Trilineage Analysis

hBMSC (P2-P4, 1 donor, 24Y male) was expanded using Minimum Essential Medium alpha (αMEM, GIBCO: 22571 – 020) supplemented with 10 % Fetal Bovine Serum (FBS, GIBCO: 10500-064), 1% Penicillin-Streptomycin 100X (SIGMA: P433) and 5 ng/ml of Basic human fibroblast growth factor (bFGF - Peprotech:100-18B-10UG). The hBMSC was cultured until 70-80% confluency. The following analysis was carried out to validate the trilineage capability.

1.1) Chondrogenesis

hBMSC (P2-P4, 24Y male) were seeded in a 1.5 ml Eppendorf tube at 100.000 cells/pellet density. After 24 hours, the medium was changed to the following media: DMEM (HG) medium and chondrogenic medium.

Here, DMEM (HG) medium consisted of Dulbecco's modified Eagle's medium (DMEM; 4.5 g/L glucose) supplemented with 10 % Fetal Bovine Serum (FBS, GIBCO: 10500-064), 1% Penicillin-Streptomycin 100X (SIGMA: P433) and 5 ng/ml of Basic human fibroblast growth factor (bFGF - Peprotech:100-18B-10UG). Further, the chondrogenic medium consisted of DMEM-HG with L-Glutamine (Gibco 11965-092), 1 % Penicillin/Streptomycin (Sigma P4333-100), 1% (v/v) ITS+ (Gibco 41400-045), 1 mM Sodium Pyruvate (Sigma S8636-100 mL), 50 µg/ml L-Proline (Sigma P5607-25 g), 1% (v/v) HEPES (Sigma H0887-20 mL or Corning 25-060-CI), 100 nM Dexamethasone (Sigma D4902-25mg), 150 µM ascorbic acid 2-phosphate (Sigma 49752), 10 ng/ml human transforming growth factor β3 (TGFβ3, Peprotech 100-36E), 100 ng/ml human bone morphogenic protein 6 (BMP-6, R&D 507-BP-020). Medium changes were carried out thrice a week, while TGFβ3 and ascorbic acid 2-phosphate were added fresh upon each medium change.

After 18 days of culture, sulphated glycosaminoglycan (GAG) and DNA were quantified. Pellets were digested using 125 µg/mL papain in 0.1 M sodium acetate, 10 mM L-cysteine-HCl, 50 mM EDTA (all from Sigma-Aldrich) adjusted pH 6.0 and incubated at 55 °C under constant shaking for 18 h. DNA content of each sample was quantified using the Quant-iT PicoGreen assay (Thermofisher, USA). The GAG content of each sample was measured (n = 3 per group and time point) using the dimethyl methylene blue dye-binding assay (DMMB, Blyscan, Biocolor Ltd., United Kingdom) and chondroitin sulphate as standards. The GAG content of each sample was normalised to the measured DNA content to receive the desired GAG/DNA ratio (n = 3 per group and time point).

1.2) Osteogenesis

hBMSC (P2-P4, 24Y male) was trypsinised and seeded in a 6-well plate at 50.000 cells/well density. After 24 hours, the medium was changed to the following media: DMEM (HG) medium and osteogenic medium.

DMEM (HG) medium was prepared as mentioned in *section 1.1*. Further, the osteogenic medium is consisting of DMEM-HG with L-Glutamine (Gibco 11965-092), 1 % Penicillin/Streptomycin (Sigma P4333-100), 10 nM Dexamethasone (Sigma D4902), 150 µM 2-Phospho-L-ascorbic acid trisodium salt (Sigma 49752), 10 mM - β-Glycerophosphate (Sigma 50020), 10 nM 1α, 25-Dihidroxyvitamin D3 (Sigma 17936). Medium changes were conducted three times a week.

On days 7 and 18, the cells were stained with Alizarin Red S (ARS) to visualize the calcium deposition. To prepare staining solution, the ARS powder (Sigma, A5533) was dissolved in 50 mL of distilled water to yield a 40 mM solution. This solution was then adjusted to pH 4.2 by adding either ammonium hydroxide or hydrochloric acid, filtered, and stored in foil-wrapped

tubes at room temperature. The cells were rinsed twice with PBS, fixed in HistoFix (ROTI ® Histofix, P087.4) for 15 minutes, and washed three times with distilled water. They were then incubated at room temperature in the ARS solution for one hour, followed by five successive water rinses. Finally, the cells were imaged using an Echo Revolve 4K microscope (Echo, San Diego, CA, USA).

1.3) Adipogenesis

hBMSC (P2-P4, 24Y male) was trypsinised and seeded in a 6-well plate at a 50,000 cells/well density. After 24 hours, the medium was changed to the following media: DMEM (HG) medium and Adipogenic medium.

DMEM (HG) medium was prepared as mentioned in *section 2.1.1*. Further, the Adipogenic medium consists of DMEM-HG with L-Glutamine (Gibco 11965-092), 1 % Penicillin/Streptomycin (Sigma P4333-100), 1 µM Dexamethasone (Sigma-D4902), 0.5 mM Isobutyl methylxanthine (IBMX, Sigma-I5879), 0.5 mM Indomethacin (Sigma-I7378), 1 µg/ml Insulin (Sigma-I9278). Medium changes were conducted three times a week.

On days 7 and 18, the cells were washed twice with PBS after removing the culture medium to prepare for staining. A working BODIPY™ 493/503 solution (Invitrogen™, D3922) was made by diluting the 3.8 mM ethanol stock into 1:1,000 in PBS. 1 ml of this staining solution was added to each well and incubated at room temperature for up to 30 minutes. After incubation, nuclei were counterstained with Hoechst (Invitrogen™, H3570) solution (1:500 in PBS) for 30 min at room temperature. Excess dye was removed by washing the cells three times with PBS. Cells were then visualised under an LSM800 confocal microscope.

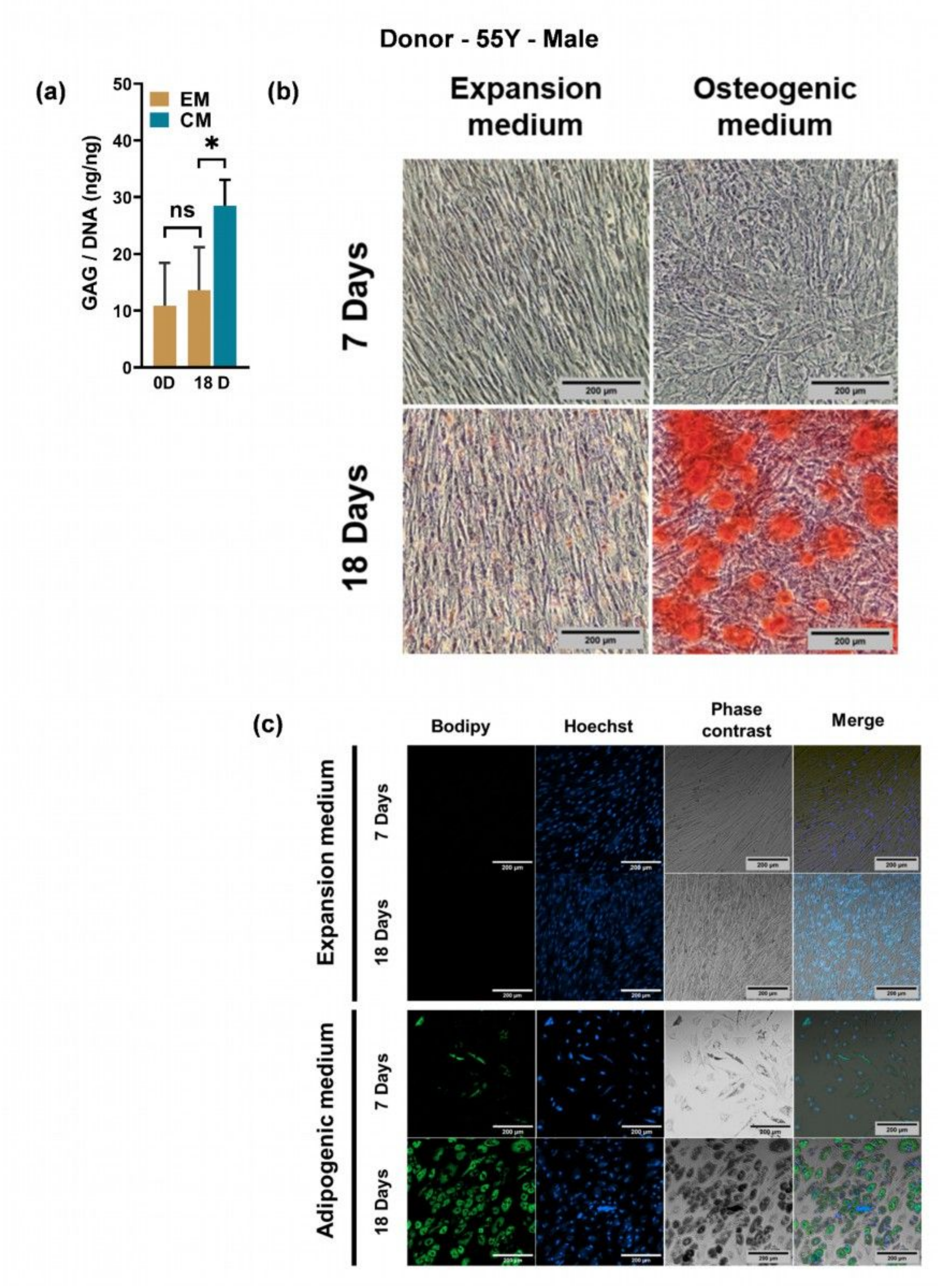

Figure S1: Trilineage analysis for hBMSC; (a) Chondrogenic lineage analysed with GAG/DNA ratio, (b) Osteogenic lineage analysed with alizarin red staining, (c) Adipogenic lineage was analysed with Bodipy staining with Hoechst as a counter stain.

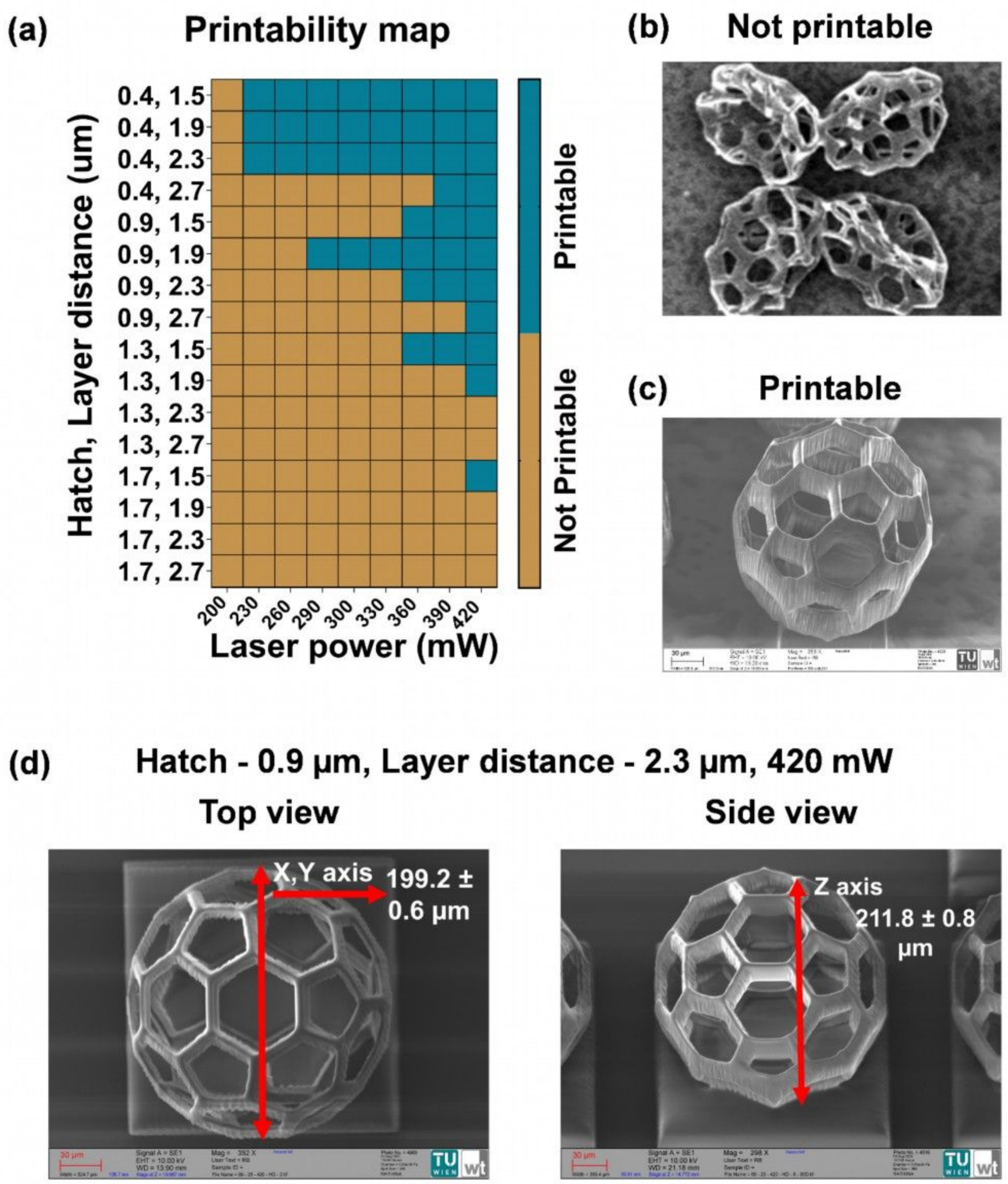

Figure S2: Optimization of printing parameter for 200 µm microscaffold; (a) Printability map shows the different parameters used to analyse based on the (b) not printable and (c) printable microscaffold; (d) SEM image shows the optimised microscaffold with various dimensions.

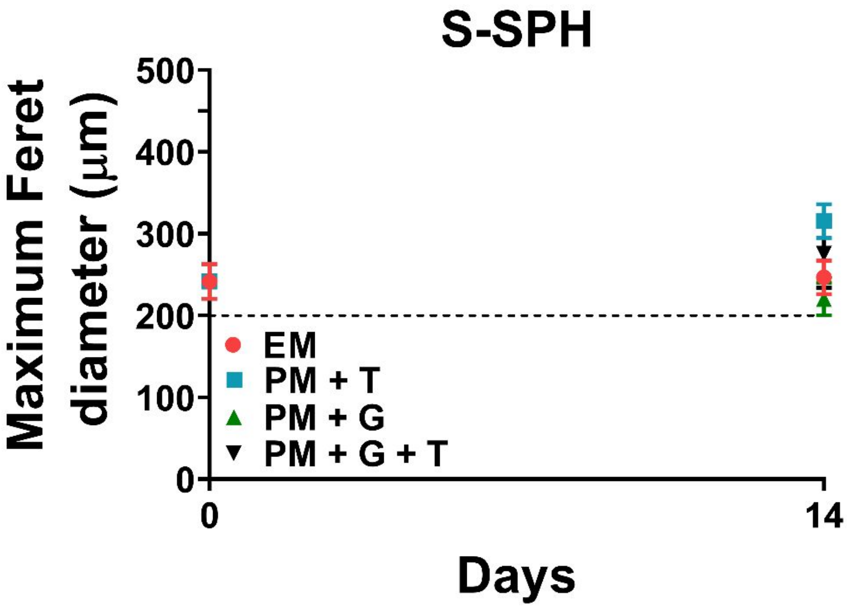

Figure S3: Feret diameter changes during the differentiation of S-SPH under different medium conditions.

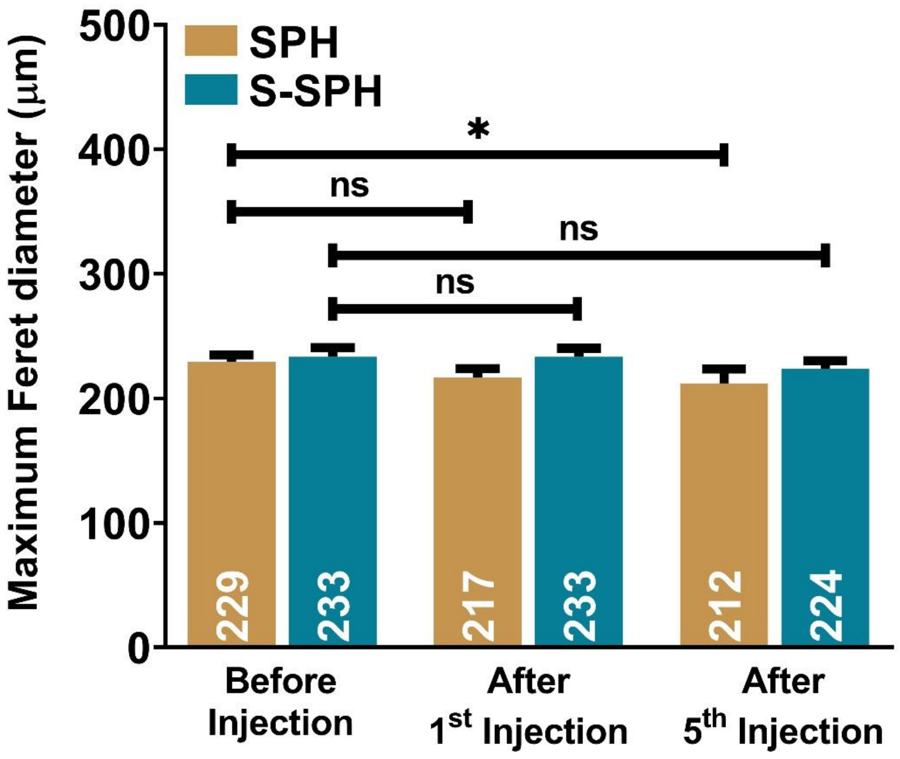

Figure S4: Effect of feret diameter changes during the injection procedure on SPH and S-SPH. * denote significance with P value <0.05 (n=3, biological replicates).
